# Supplementary figures and images for: Risk of coronary heart disease in the rural population in Xinjiang: A nested case-control study in China
Source: PLoS One. 2020 Mar 4;15(3):e0229598. doi: 10.1371/journal.pone.0229598 (PMC7055895; doi:10.1371/journal.pone.0229598)

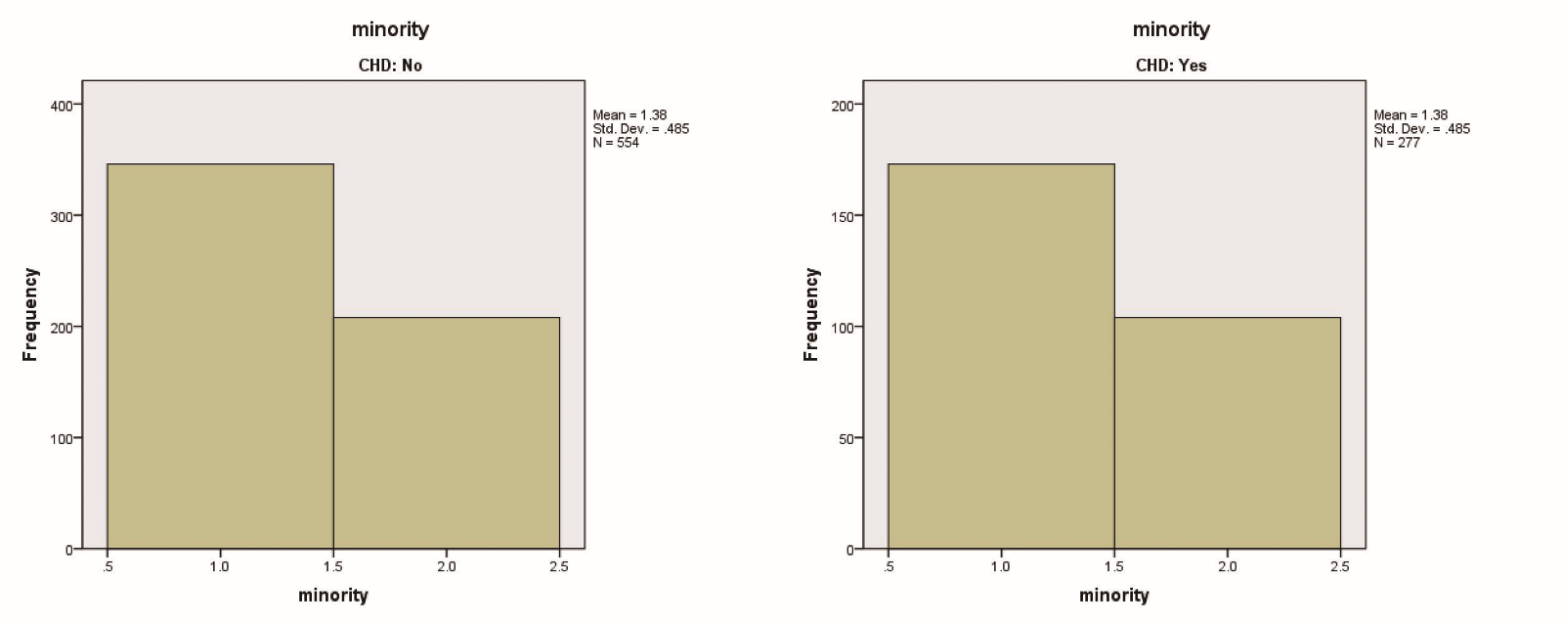


**Figure S1.** Histogram of minority between Case and Control by SPSS

Supplement: S1 Fig — (DOCX) [file pone.0229598.s001.docx]

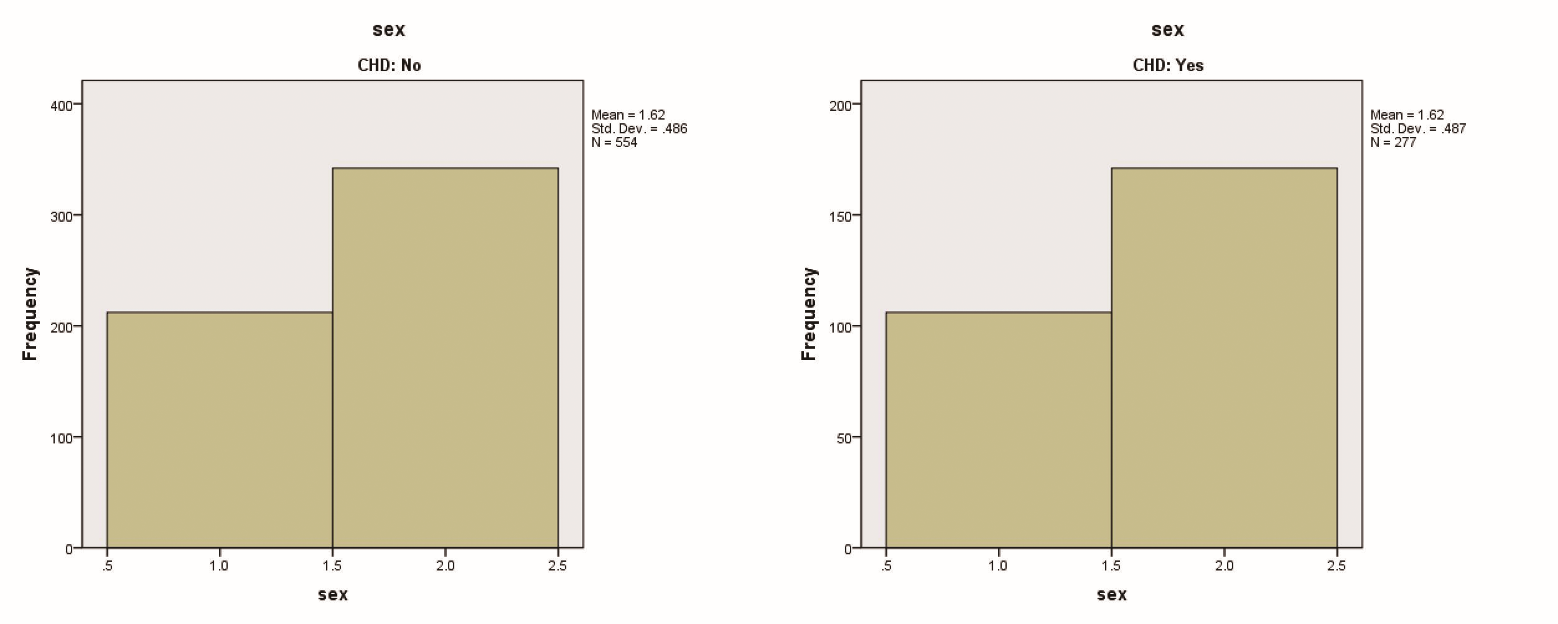


**Figure S2.** Histogram of sex between Case and Control by SPSS

Supplement: S2 Fig — (DOCX) [file pone.0229598.s002.docx]

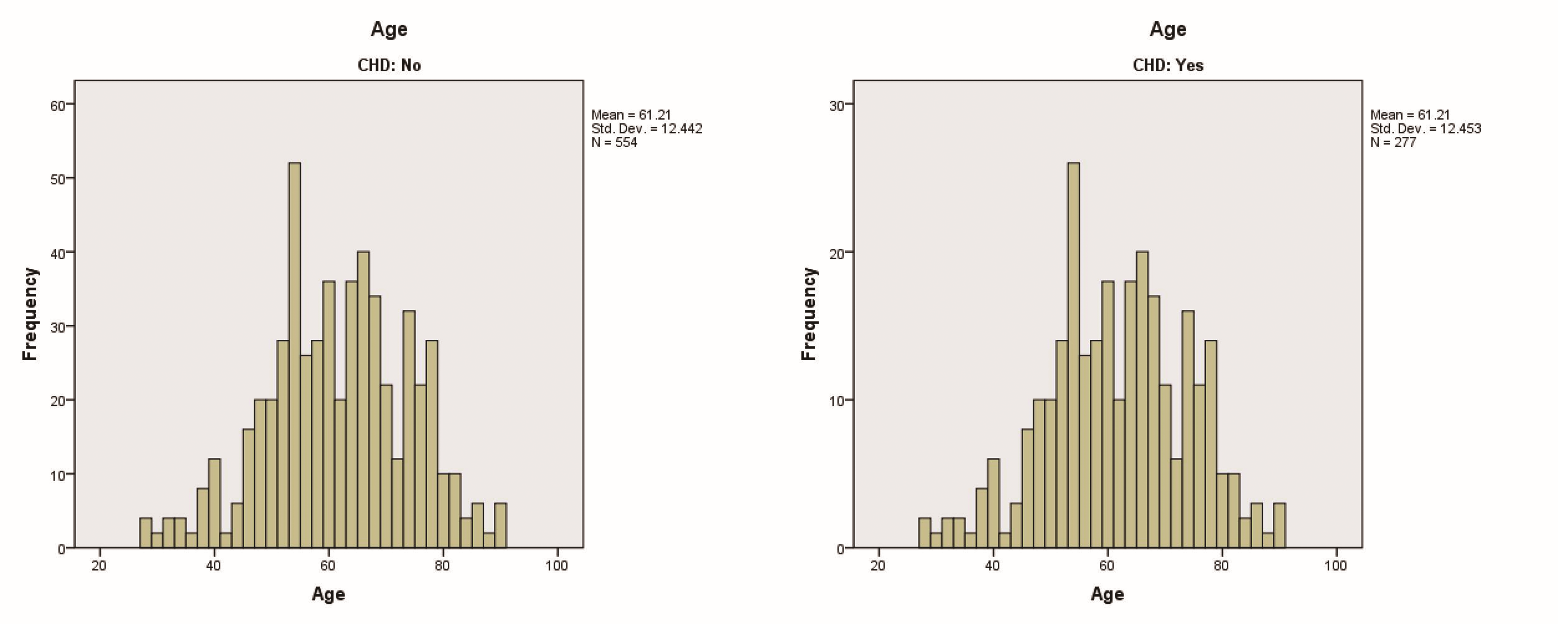


**Figure S3.** Histogram of age between Case and Control by SPSS

Supplement: S3 Fig — (DOCX) [file pone.0229598.s003.docx]
