# Supplementary material for: Risk of coronary heart disease in the rural population in Xinjiang: A nested case-control study in China
Source: PLoS One. 2020 Mar 4;15(3):e0229598. doi: 10.1371/journal.pone.0229598 (PMC7055895; doi:10.1371/journal.pone.0229598)
Supplement: S1 Table — (DOCX) [file pone.0229598.s004.docx]

| **Table S1.** Description of matching factors between Case and Control by SPSS (minority) | | | |
| --- | --- | --- | --- |
| **CHD** | **Minority** | **Frequency (n)** | **Percent of sample (%)** |
| No | Kazakhs | 346 | 62.5 |
|  | Uyghurs | 208 | 37.5 |
|  | Total | 554 | 100.0 |
| Yes | Kazakhs | 173 | 62.5 |
|  | Uyghurs | 104 | 37.5 |
|  | Total | 277 | 100.0 |
