# Supplementary material for: Risk of coronary heart disease in the rural population in Xinjiang: A nested case-control study in China
Source: PLoS One. 2020 Mar 4;15(3):e0229598. doi: 10.1371/journal.pone.0229598 (PMC7055895; doi:10.1371/journal.pone.0229598)
Supplement: S2 Table — (DOCX) [file pone.0229598.s005.docx]

| **Table S2.** Description of matching factors between Case and Control by SPSS (sex) | | | |
| --- | --- | --- | --- |
| **CHD** | **Sex** | **Frequency (n)** | **Percent of sample (%)** |
| No | Male | 212 | 38.3 |
|  | Female | 342 | 61.7 |
|  | Total | 554 | 100.0 |
| Yes | Male | 106 | 38.3 |
|  | Female | 171 | 61.7 |
|  | Total | 277 | 100.0 |
