# Supplementary material for: Risk of coronary heart disease in the rural population in Xinjiang: A nested case-control study in China
Source: PLoS One. 2020 Mar 4;15(3):e0229598. doi: 10.1371/journal.pone.0229598 (PMC7055895; doi:10.1371/journal.pone.0229598)
Supplement: S3 Table — (DOCX) [file pone.0229598.s006.docx]

| **Table S3.** Description of matching factors between Case and Control by SPSS (age) | | | |
| --- | --- | --- | --- |
| **CHD** | **N** | **Mean (years)** | **Std. Deviation (years)** |
| No | 554 | 61.21 | 12.442 |
| Yes | 277 | 61.21 | 12.453 |
