# Supplementary material for: Risk of coronary heart disease in the rural population in Xinjiang: A nested case-control study in China
Source: PLoS One. 2020 Mar 4;15(3):e0229598. doi: 10.1371/journal.pone.0229598 (PMC7055895; doi:10.1371/journal.pone.0229598)
Supplement: S1 Questionnaire — (DOCX) [file pone.0229598.s007.docx]

**调查问卷**

1. ID 个人编码 [填空题] *

B+四位数字

_________________________________

2. A1 姓名 [填空题] *

_________________________________

3. A2 性别： [单选题] *

| ○男 |
| --- |
| ○女 |

4. A3 你所居住的村是:？ [单选题] *

| ○拜克托别 |
| --- |
| ○喀拉奥依 |
| ○阿尔善 |
| ○恰勒阔德 |

5. A4 身份证号码： [填空题] *

_________________________________

6. G 此人调查次数 [单选题] *

| ○0 |
| --- |
| ○1 |
| ○2 |
| ○3 |
| ○4 |

7. A5 电话号码 [填空题] *

_________________________________

8. A6 出生日期： [填空题] *

_________________________________

9. A7 在本地居住年限： [单选题] *

| ○1年～ |
| --- |
| ○5年～ |
| ○10年～ |
| ○15年～ |

10. A8 民族： [单选题] *

| ○哈族 |
| --- |
| ○维族 |
| ○汉族 |
| ○蒙古族 |
| ○其他 |

11. A9 文化程度： [单选题] *

| ○文盲或半文盲 |
| --- |
| ○小学 |
| ○初中 |
| ○高中 |
| ○中专 |
| ○大专及以上 |

12. A10 职业： [单选题] *

| ○牧业 |
| --- |
| ○农业 |
| ○一般工作人员 |
| ○离退休或家务 |
| ○半农半牧 |

13. A11 婚姻状况： [单选题] *

| ○已婚 |
| --- |
| ○未婚 |
| ○离婚 |
| ○丧偶 |
| ○分居 |

14. A12 平均年收入：总收入/人口总数（包括小孩） [填空题] *

_________________________________

15. B 你是否患有下列疾病？

B0.无 B1.高血压 B2.糖尿病 B3.冠心病 B4.高脂血症

B5.结核病 B6.慢支/肺气肿 B7.慢性胃肠炎 B8.慢性肝炎(乙肝) B9.脑卒中 B10.慢性肾病 B11.心肌梗死 B12.癌症（肿瘤）B13. 肺心病 [单选题] *

| ○否 (请跳至第43题) |
| --- |
| ○是 |

16. B1 你是否患有高血压？ [单选题] *

| ○否 (请跳至第18题) |
| --- |
| ○是 |

17. B11 你确诊高血压的时间是？ [填空题]

_________________________________

18. B2 你是否患有糖尿病？ [单选题] *

| ○否 (请跳至第20题) |
| --- |
| ○是 |

19. B21 你确诊糖尿病的时间是？ [填空题]

_________________________________

20. B3 你是否患有冠心病？ [单选题] *

| ○否 (请跳至第22题) |
| --- |
| ○是 |

21. B31 你确诊冠心病的时间是？ [填空题]

_________________________________

22. B4 你是否患有高脂血症？ [单选题] *

| ○否 (请跳至第24题) |
| --- |
| ○是 |

23. B41 你确诊高脂血症的时间是？ [填空题]

_________________________________

24. B5 你是否患有结核病？ [单选题] *

| ○否 (请跳至第26题) |
| --- |
| ○是 |

25. B51 你确诊结核病的时间是？ [填空题]

_________________________________

26. B6 你是否患有慢支/肺气肿？ [单选题] *

| ○否 (请跳至第28题) |
| --- |
| ○是 |

27. B61 你确诊慢支/肺气肿的时间是？ [填空题]

_________________________________

28. B7 你是否患有慢性胃肠炎？ [单选题] *

| ○否 (请跳至第30题) |
| --- |
| ○是 |

29. B71 你确诊慢性胃肠炎的时间是？ [填空题]

_________________________________

30. B8 你是否患有慢性肝炎（乙肝）？ [单选题] *

| ○否 (请跳至第32题) |
| --- |
| ○是 |

31. B81 你确诊慢性肝炎（乙肝）的时间是？ [填空题]

_________________________________

32. B9 你是否患有脑卒中？ [单选题] *

| ○否 (请跳至第34题) |
| --- |
| ○是 |

33. B91 你确诊患脑卒中的时间是？ [填空题]

_________________________________

34. B10 你是否患有慢性肾病？ [单选题] *

| ○否 (请跳至第36题) |
| --- |
| ○是 |

35. B101 你确诊慢性肾病的时间是？ [填空题]

_________________________________

36. B11 你是否有出现过心机梗死？ [单选题] *

| ○否 (请跳至第38题) |
| --- |
| ○是 |

37. B111 你确诊心机梗死的时间是？ [填空题]

_________________________________

38. B14 你是否患有癌症（肿瘤）？ [单选题] *

| ○否 (请跳至第41题) |
| --- |
| ○是 |

39. B141 你所患癌症的名称是？ [填空题]

_________________________________

40. B141 你确诊癌症（肿瘤）的时间是？ [填空题] *

_________________________________

41. B 15 你是否患有肺心病？ [单选题] *

| ○否 (请跳至第43题) |
| --- |
| ○是 |

42. B 151 你确诊肺心病的时间是？ [填空题]

_________________________________

43. B16 你是否患有其他慢性疾病？ [填空题]

_________________________________

44. B17 你是否有过下列症状：B171 突然发作剧烈而持久的胸骨后或心前区压榨性疼痛 B172 呼吸困难、咳嗽、发绀 B173 面色苍白，皮肤湿冷，烦躁不安或神志淡漠 B174 头晕、胸闷、呼吸困难？ [单选题] *

| ○否 (请跳至第46题) |
| --- |
| ○是 |

45. B18 你首次出现上述症状的时间是？ [填空题]

_________________________________

46. B19 你是否服用硝酸甘油？ [单选题] *

| ○否 |
| --- |
| ○是 |

47. D1.高血压 [多选题] *

| □父母均有 |
| --- |
| □父亲有 |
| □母亲有 |
| □兄弟姐妹有 |
| □无 |

48. D2.糖尿病 [多选题] *

| □父母均有 |
| --- |
| □父亲有 |
| □母亲有 |
| □兄弟姐妹有 |
| □无 |

49. D3.是否有癌症 [多选题] *

| □父母均有 |
| --- |
| □父亲有 |
| □母亲有 |
| □兄弟姐妹有 |
| □无 |

50. D31 患有癌症的名称是： [填空题]

_________________________________

51. D5.脑卒中 [多选题] *

| □父母均有 |
| --- |
| □父亲有 |
| □母亲有 |
| □兄弟姐妹有 |
| □无 |

52. D6.冠心病 [多选题] *

| □父母均有 |
| --- |
| □父亲有 |
| □母亲有 |
| □兄弟姐妹有 |
| □无 |

53. D9 你的父母或者兄弟姐妹是否患有其他疾病？ [单选题] *

| ○否 (请跳至第55题) |
| --- |
| ○是 |

54. D91 你的父母或者兄弟姐妹患有的其他疾病名称是？ [填空题]

_________________________________

55. F1 您吸烟吗？ [单选题] *

| ○否 (请跳至第59题) |
| --- |
| ○吸烟 (请跳至第57题) |
| ○已戒烟 |

56. F2 如您已戒烟，戒烟多少年了（写具体年数）： [填空题]

_________________________________

57. F3 如您曾吸烟，开始吸烟的年龄： [填空题]

_________________________________

58. F5 您每天吸烟量： [单选题]

| ○半包以内 |
| --- |
| ○半包至一包 |
| ○一包以上 |

59. G1您是否饮酒： [单选题] *

| ○否 (请跳至第65题) |
| --- |
| ○是 (请跳至第61题) |
| ○已戒酒 |

60. G2 您多大年龄停止饮酒的： [填空题]

_________________________________

61. G3 您多大年龄开始养成每周都喝酒的习惯： [填空题]

_________________________________

62. G4您平均每周大约喝几次酒： [单选题] *

| ○1-2次 |
| --- |
| ○3-4次 |
| ○每周5次以上 |

63. G5 饮酒种类： [单选题] *

| ○酒精度小于 15% |
| --- |
| ○15%～ |
| ○30%～ |
| ○大于45% |

64. G6 您每次喝酒的习惯（1杯相当于50ml）： [单选题] *

| ○1-2杯 |
| --- |
| ○3-4杯 |
| ○5-6杯 |
| ○7-8杯 |
| ○8杯及以上 |

65. H1 您是否饮奶茶： [单选题] *

| ○否 (请跳至第68题) |
| --- |
| ○饮 |

66. H2 您通常是喜欢喝盐淡的还是盐重的奶茶： [单选题]

| ○盐淡 |
| --- |
| ○适度 |
| ○盐重 |

67. H4 您通常一天喝几碗奶茶？（碗/天） [填空题]

_________________________________

68. 您家通常每月食盐消耗量：_________    （g/月）______口人吃
大人：_______________小孩_________（7岁及以下） [填空题] *

69. I5您是否吃熏马（肠、肉）： [单选题] *

| ○否 (请跳至第71题) |
| --- |
| ○是 |

70. I6您通常每年吃多少公斤熏马（肠、肉）: （公斤/年）一匹马150公斤 [填空题]

_________________________________

71. I7 你是否吃腌肉 [单选题] *

| ○否 (请跳至第73题) |
| --- |
| ○是 |

72. I8 您通常每年吃多少公斤腌肉: （公斤/年）一只羊30公斤 [填空题]

_________________________________

73. I9 鲜奶（1份200-250ml，相当于1碗）： [单选题] *

| ○不喝或每周少于1份 |
| --- |
| ○每周1-3份 |
| ○每周4-6份 |
| ○每天1份 |
| ○每天2份及以上 |

74. I10 奶制品（1份相当于30g奶酪）： [单选题] *

| ○不吃或每周少于1份 |
| --- |
| ○每周1-3份 |
| ○每周4-6份 |
| ○每天1份 |
| ○每天2份及以上 |

75. I11蛋类（1份相当于1个鸡蛋，）： [单选题] *

| ○不吃或每周少于1份 |
| --- |
| ○每周1-3份 |
| ○每周4-6份 |
| ○每天1份 |
| ○每天2份及以上 |

76. I12新鲜肉： [单选题] *

| ○不吃或每月少于1公斤 |
| --- |
| ○每月1公斤到2公斤 |
| ○每月2公斤及以上 |

77. I13内脏类（肝、肠、心、肾、鸡胗等1份100g）： [单选题] *

| ○不吃或每周少于1份 |
| --- |
| ○每周1-3份 |
| ○每周4-6份 |
| ○每天1份 |
| ○每天2份及以上 |

78. I14蔬菜（使用16cm口径盘）： [单选题] *

| ○不吃或每天少于半盘 |
| --- |
| ○每天半盘-1盘 |
| ○每天1-1.5盘 |
| ○每天1.5-2盘 |
| ○每天2盘及以上 |

79. I15 用动物油炒菜 [单选题] *

| ○不用 |
| --- |
| ○每周1-2次 |
| ○每周3-4次 |
| ○每周5-6次 |
| ○每周7次以上 |

80. I16新鲜水果（1份相当于1个苹果或梨子或5个杏子）： [单选题] *

| ○不吃或每周少于1份 |
| --- |
| ○每周1-3份 |
| ○每周4-6份 |
| ○每天1份 |
| ○每天2份及以上 |

81. I17面食（1份相当于半块馕或1盘拉面）： [单选题] *

| ○每天1份 |
| --- |
| ○每天2份 |
| ○每天3份 |
| ○每天4份 |
| ○5份及以上 |

82. I18抓饭(1份相当于1碗（8cm口径)或1盘（16cm口径））： [单选题] *

| ○不吃或每周少于1份 |
| --- |
| ○每周1-3份 |
| ○每周4-6份 |
| ○每天1份 |
| ○每天2份及以上 |

83. I19 油炸食物： [单选题] *

| ○不吃或每周少于1次 |
| --- |
| ○每周1-3次 |
| ○每周4-6次 |
| ○每天1次 |
| ○每天2次及以上 |

84. I20 酥油： [单选题] *

| ○无 |
| --- |
| ○每天1-2勺 |
| ○每天3-4勺 |
| ○每天5-6勺 |
| ○6勺及以上 |

85. J1 你平时工作性质： [单选题] *

| ○放牧 |
| --- |
| ○务农 (请跳至第88题) |
| ○家务 (请跳至第88题) |
| ○非放牧/务农 (请跳至第88题) |

86. J2 每年放牧时间？（精确到月） [填空题]

_________________________________

87. J3 每天放牧时间几小时： [填空题]

_________________________________

88. M1 颈围（cm）？保留1位小数 [填空题] *

_________________________________

89. M2 身高(cm)保留1位小数： [填空题] *

_________________________________

90. M3 体重(kg)保留1位小数： [填空题] *

_________________________________

91. M4 体质指数 [填空题] *

_________________________________

92. M5 身体脂肪率 [填空题] *

_________________________________

93. M6 内脏脂肪指数 [填空题] *

_________________________________

94. M7 基础代谢率 [填空题] *

_________________________________

95. M8 腰围(cm)： [填空题] *

_________________________________

96. M9 臀围(cm)： [填空题] *

_________________________________

97. M101 第一次血压收缩压：_________  舒张压：_________ [填空题] *

98. M102 第二次血压收缩压：_________    舒张压：___ [填空题] *

99. M103 第三次血压收缩压：_________    舒张压：_________ [填空题]
