# Supplementary material for: Risk of coronary heart disease in the rural population in Xinjiang: A nested case-control study in China
Source: PLoS One. 2020 Mar 4;15(3):e0229598. doi: 10.1371/journal.pone.0229598 (PMC7055895; doi:10.1371/journal.pone.0229598)
Supplement: Attachment — Submitted filename: Reviewer_response_PLOSone_2019-09-13.docx [file pone.0229598.s009.docx]

**Dear manuscript authors,**

This paper presents the potential interactions among the main risk factors of CHD. It helps to clarify the risk factors of CHD for the population in Xinjiang.

This study presents some original research and they are important only for this region. Some definitions, for example, obesity, overweight are different compared to WHO recommendations.

Some question from the Methodology chapter:

1. What criteria were used to match the cases and control groups? You wrote, "*matched by propensity score matching*". Please describe the factors that have been involved, and indicate the sources of literature where this sampling method would be used or described. Is age, gender, education, etc. taken into account when matching the groups?
2. Please explain in more detail what kind of questionnaires (24h-recall, FFQ, etc.) have been used to assess dietary habits.

A high-fat diet needs to be described in more detail. How was this diet calculated? How much energy per day from fats received responders? What kind of criteria were used to identify the high-fat diet of respondents? It is also necessary to refer to the sources of literature.

It is extremely important to describe this because you have obtained meaningful results and presented them in the conclusions.

3. It is necessary to provide literature references or recommendations that you have used to evaluate overweight, obesity, and the criteria for WC and dyslipidemias.

Results section

In Table 1 you presented, that only total cholesterol values significantly differed in cases compared to controls, however, neither did TG, LDL-c, or HDL-C, but the incidence of dyslipidemias was significantly different. How do you explain this?

Are the data in this table adjusted for gender and age?

Please provide the full p values in Table 2. In my opinion, some presented data are not significantly different.

Discussion section.

More attention should be paid to the interaction between risk factors for CHD and to comparisons these results with researches in other countries. On the other hand, it is difficult for the reader to compare those results because of the different evaluation criteria and the uncertainty of what a high-fat diet represents (EU is considered to be a high-fat diet if the daily energy from fat is more than 65% per day).

The conclusion is suitable and reflects on the aim.

Literature should be updated with more recent sources, as only 40% of sources are under 5 years old.

The English version of the article should be improved.
